# Biomechanical evaluation of a novel anterior transpedicular screw-plate system for anterior cervical corpectomy and fusion (ACCF): a finite element analysis

**DOI:** 10.3389/fbioe.2023.1260204

**Published:** 2023-11-09

**Authors:** Shengbin Huang, Qinjie Ling, Xinxin Lin, Hao Qin, Xiang Luo, Wenhua Huang

**Affiliations:** ^1^ Department of Human Anatomy, School of Basic Medical Sciences, Guangxi Medical University, Nanning, Guangxi, China; ^2^ Department of Orthopedics, The Eighth Affiliated Hospital of Guangxi Medical University, Guigang, Guangxi, China; ^3^ Department of Spinal Surgery, The First Affiliated Hospital of Guangzhou Medical University, Guangzhou, Guangdong, China; ^4^ National Key Discipline of Human Anatomy, Guangdong Provincial Key Laboratory of Medical Biomechanics, Guangdong Engineering Research Center for Translation of Medical 3D Printing Application, School of Basic Medical Sciences, Southern Medical University, Guangzhou, Guangdong, China

**Keywords:** cervical spine, anterior pedicle screw, anterior surgery, corpectomy, reconstruction, biomechanics, finite element analysis

## Abstract

**Background and objective:** Cervical fusion with vertebral body screw (VBS)-plate systems frequently results in limited biomechanical stability. To address this issue, anterior transpedicular screw (ATPS) fixation has been developed and applied preliminarily to multilevel spinal fusion, osteoporosis, and three-column injury of the cervical spine. This study aimed to compare the biomechanical differences between unilateral ATPS (UATPS), bilateral ATPS (BATPS), and VBS fixation using finite element analysis.

**Materials and methods:** A C6 corpectomy model was performed and a titanium mesh cage (TMC) and bone were implanted, followed by implantation of a novel ATPS-plate system into C5 and C7 to simulate internal fixation with UATPS, BATPS, and VBS. Internal fixation with UATPS comprises ipsilateral transpedicular screw-contralateral vertebral body screw (ITPS-CVBS) and cross transpedicular screw-vertebral body screw (CTPS-VBS) fixations. Mobility, the maximal von Mises stress on TMC, the stress distribution and maximal von Mises stress on the screws, and the maximum displacement of the screw were compared between the four groups.

**Results:** Compared with the original model, each group had a reduced range of motion (ROM) under six loads. After ACCF, the stress was predominantly concentrated at two-thirds of the length from the tail of the screw, and it was higher on ATPS than on VBS. The stress of the ATPS from the cranial part was higher than that of the caudal part. The similar effect happened on VBS. The screw stress cloud maps did not show any red areas reflective of a concentration of the stress on VBS. Compared with VBS, ATPS can bear a greater stress from cervical spine movements, thus reducing the stress on TMC. The maximal von Mises stress was the lowest with bilateral transpedicular TMC and increased with cross ATPS and with ipsilateral ATPS. ITPS-CVBS, CTPS-VBS, and BATPS exhibited a reduction of 2.3%–22.1%, 11.9%–2.7%, and 37.9%–64.1% in the maximum displacement of screws, respectively, compared with that of VBS.

**Conclusion:** In FEA, the comprehensive stability ranked highest for BATPS, followed by CTPS-VBS and ITPS-CVBS, with VBS demonstrating the lowest stability. Notably, utilizing ATPS for fixation has the potential to reduce the occurrence of internal fixation device loosening after ACCF when compared to VBS.

## 1 Introduction

Degeneration, trauma, and infection of the lower cervical spine frequently occur in the anterior column, and conventional anterior fixation with plates and screws is usually used in most cases undergoing diskectomies or corpectomis. In patients with osteoporosis or those requiring multilevel decompression and reconstruction, fixation with vertebral body screw (VBS)-plate systems frequently results in limited biomechanical stability and loosening of internal fixation devices (Singh et al., 2004). Koller et al. (2007) reviewed the literature and found the non-fusion rate of multilevel anterior cervical discectomy and fusion (ACDF) to be 20%–50% and the failure rate of anterior cervical corpectomy and fusion (ACCF) to be 30%–100%. Bayerl et al. (2019) retrospectively analyzed 21 patients who underwent two-level cervical corpectomy, and long-term postoperative follow-ups revealed that the instability rate was up to 33% after fixation only with the anterior VBS system. Hence, they recommended additional posterior spinal fusion after two-level cervical corpectomy to increase the stability of anterior fixation and reduce the failure rate and complications of surgery. However, additional posterior surgery not only increases the economic burden, but also increases surgical complications (Okawa et al., 2011; Mushkin et al., 2019).

Pedicle screw fixation can offer adequate stability of the cervical spine (Henriques et al., 2015). Biomechanical research show that ATPS performs significantly better than VBS (Koller et al., 2008a; Wu et al., 2015). Koller et al. (2008b) demonstrated that the ATPS technique for the cervical spine takes advantage of both anterior and posterior approaches and can prevent loosening of internal fixation devices. In addition, this technique can overcome the inadequacy of fixation strength of VBS in patients with osteoporosis and, thus, results in enhanced biomechanical stability. In clinical practice, the ATPS-plate system is rarely available and it is difficult to insert bilateral ATPS (BATPS) into the lower cervical spine because of the hindrance of the trachea and esophagus and the lack of other factors such as the computer navigation systems. Consequently, there are few reports of the clinical application of the ATPS fixation technique for the cervical spine. There have been many reports of unilateral transpedicular screw fixation (Aramomi et al., 2008; Yukawa et al., 2009; Ikenaga et al., 2012) or fixation with unilateral ATPS (UATPS) plus VBS (Zhang et al., 2016). However, there is paucity of literature on the differences in the stability of UATPS and BATPS for the cervical spine, and on comparative biomechanical advantages of different orientations of unilateral screws.

The action forces among the vertebral bodies of the cervical spine and their surrounding muscles and ligaments are complicated, and both animal and cadaver models have drawbacks. Hence, biomechanical finite element analysis (FEA) of the cervical spine, as a supplement to animal and cadaver studies, has been widely used. It is a tool for predicting and preparing for clinical trials. After the finite element test predictions are reasonable, *in vitro* experiments need to be conducted for analysis and verification before clinical trials. The finite element method allows for the establishment of three-dimensional (3D) finite element models for specific scenarios and facilitates analysis of the efficacy of various therapeutic regimens (Biswas et al., 2018; Dai et al., 2022). Finite element models can also determine the engineering basis for device design and provide technical recommendations (Ling et al., 2019). This study aimed to explore the biomechanical differences between UATPS (two screw orientations), BATPS, and VBS by testing a novel ATPS-plate system for the cervical spine through finite element analysis, so as to provide a theoretical basis for the clinical use of ATPS for the cervical spine.

## 2 Materials and methods

### 2.1 Construction of C3–C7 finite element models

The study subject was a 32-year-old healthy male volunteer. This study was approved by the Ethics Committee of Guangxi Medical University, and informed consent was obtained from the volunteer. A 3D finite element model of C3–C7 was reconstructed with computed tomography (CT) data using Mimics 20.0 (Materialise, Leuven, Belgium). Next, the 3D model was smoothed and polished using Geomagic 12.0 (Geomagic, United States). In the model, the cortical and cancellous bone, endplate, annulus fibrosus, nucleus pulposus, posterior elements, anterior longitudinal ligament, posterior longitudinal ligament, capsular ligament, transverse ligament, ligamentum flavum, interspinous ligament, supraspinous ligament and capsular ligament were reconstructed. Table 1 lists all the material properties and element types of these tissues according to Polikeit et al. (2003).

**TABLE 1:** Parameters of the various tissues of the cervical spine (Polikeit et al., 2003).

Structure	Young’s modulus (MPa)	Modulus (MPa) Poisson’s ratio	Area (mm^2^)	Element type
Cortical bone	12,000	0.3		solid186
Cancellous bone	100	0.2		solid186
Endplate	1,000	0.4		solid186
Posterior elements	3,500	0.25		solid186
Nucleus pulposus	0.2	0.4999		solid186
Annulus fibrosus	4.2	0.45		solid186
Anterior longitudinal ligament	20	0.3	38	link180
Posterior longitudinal ligament	70	0.3	20	link180
Ligamentum flavum	50	0.3	60	link180
Interspinal ligament	28	0.3	35.5	link180
Supraspinous ligament	28	0.3	35.5	link180
Capsular ligament	20	0.3	40	link180
Titanium prosthesis	116,000	0.3		solid186

**FIGURE 1:**
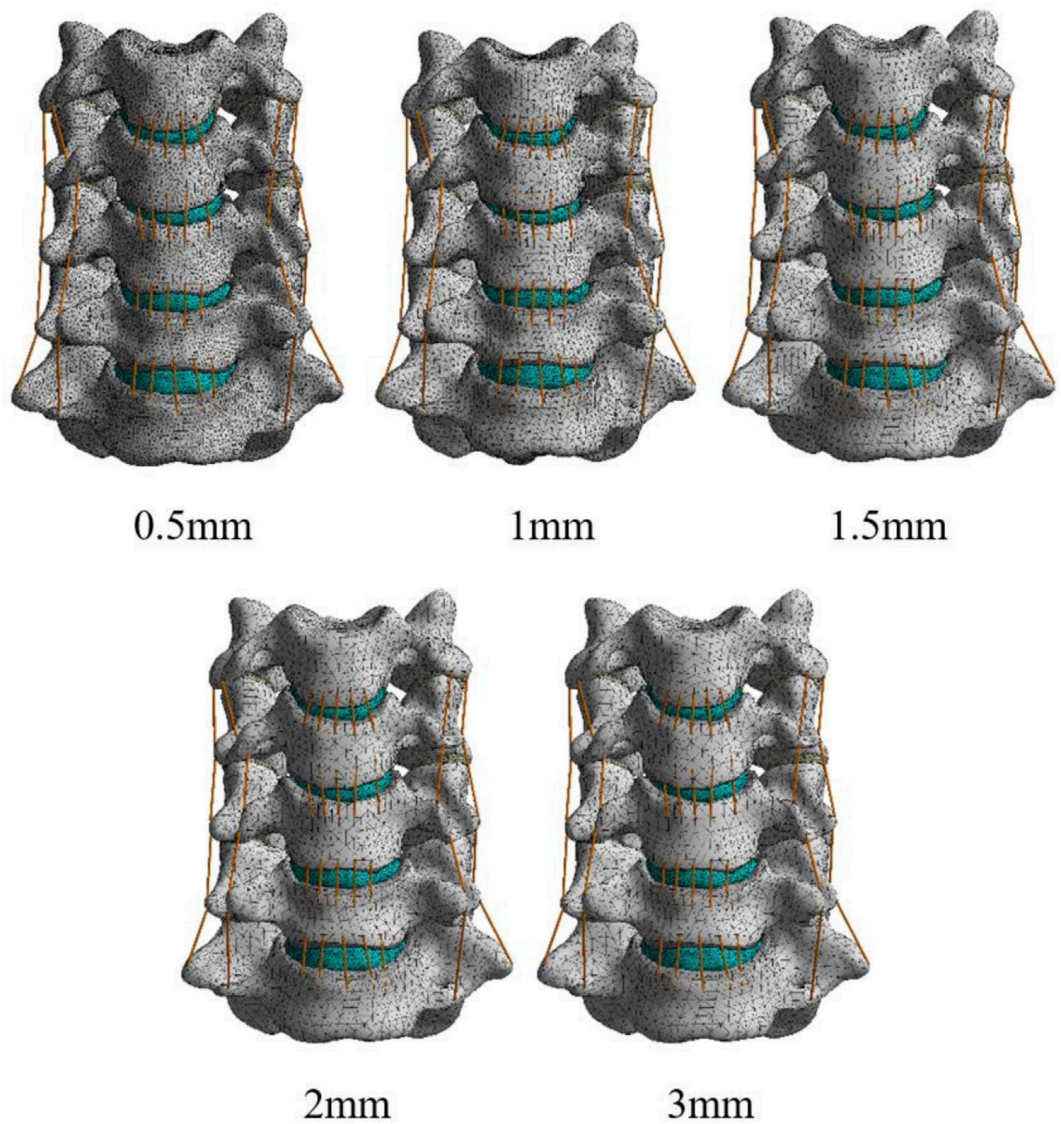
The mesh FE model.

**FIGURE 2:**
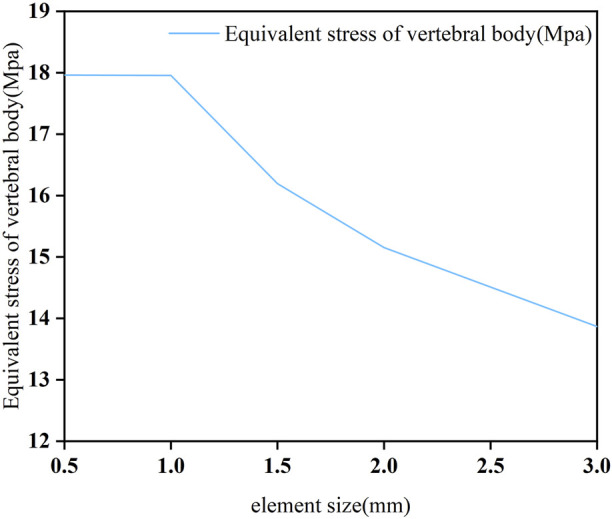
Mesh convergence analysis.

To obtain accurate data, the mesh of the model was validated. The mesh convergence test was performed with the 3D finite element model of C3–C7, and the mesh was divided by five sizes (0.5, 1, 1.5, 2, and 3 mm) (Figure 1). The five mesh models were subjected to testing, with the maximal von Mises stress on the vertebral body being the parameter of interest. Figure 2 shows the relationship between the stress and the mesh size. Based on the calculation time and results, the requirement of a change rate of <5% for the maximal von Mises stress was met (Dai et al., 2022). Hence, the unit size of 1 mm was used as the final mesh size for this study (Table 2).

**TABLE 2:** Mesh convergence test results.

Mesh size (mm)	Nodes	Units	Change rate of the maximal von Mises stress (%)
0.5	865,134	574,841	-
1	235,972	374,193	<5
1.5	214,571	156,216	>5
2	177,387	125,396	>5
3	143,318	110,125	>5

### 2.2 Boundary and loading conditions of FE models

**FIGURE 3:**
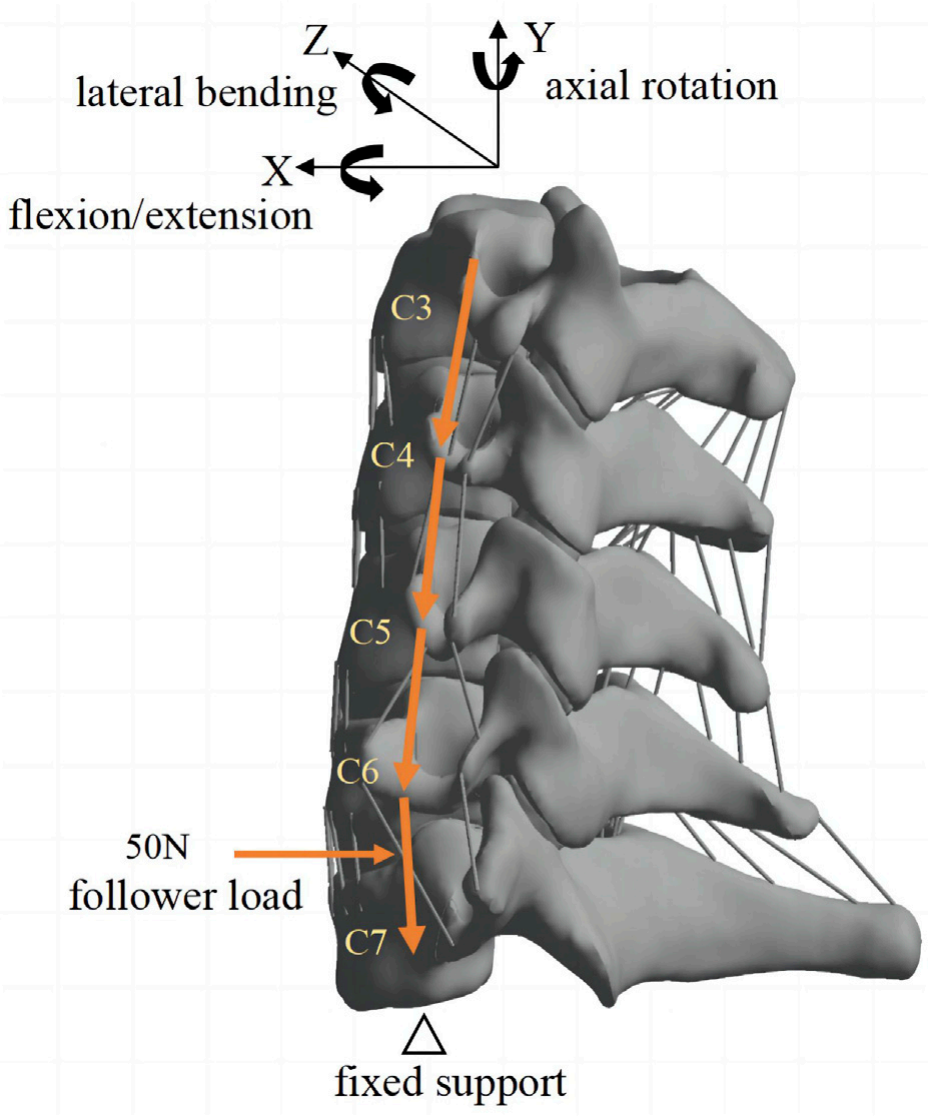
Loading and boundary conditions of the C3–C7 cervical model.

Setup of the C3–C7 model (Figure 3): All facet joints were set as contact, with a friction co-efficient of 0.1 (Liu et al., 2011). All degrees of freedom of the endplate beneath C7 were restricted, and a pre-load of 50 N was applied to the endplate above C3 to simulate the weight of the head, and the additional bending moment of motion was 1 Nm (Lee et al., 2011). To validate the C3–C7 finite element model, a bending moment of 1.5 Nm was applied to the C3 plane, and the model was loaded in flexion, extension, lateral bending, and axial rotation (Lee et al., 2011), followed by calculation, data extraction, and determination of the range of motion (ROM).

### 2.3 Construction of ACCF finite element model by four types of fixation

**FIGURE 4:**
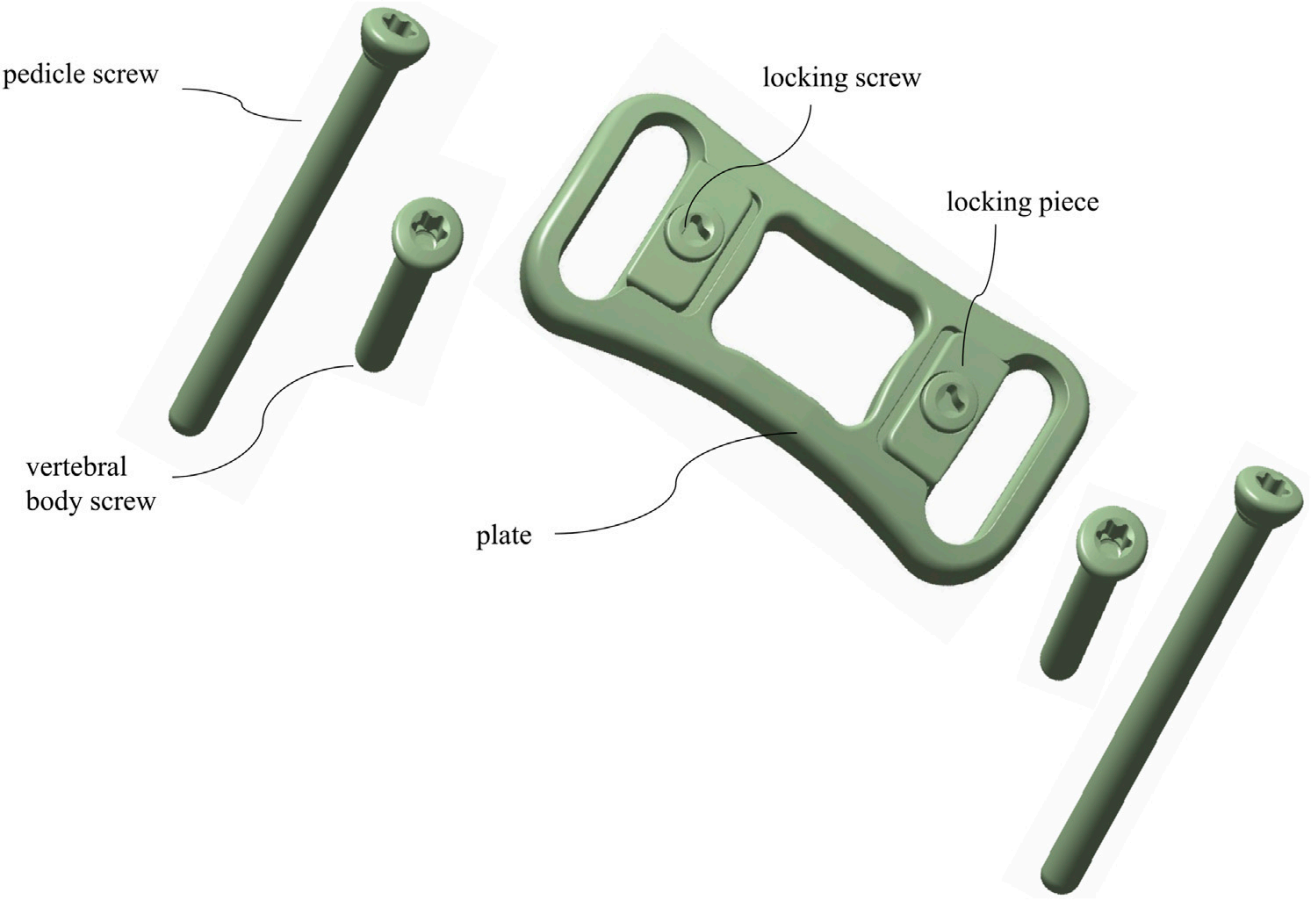
The novel anterior transpedicular screw plate systems.

**FIGURE 5:**
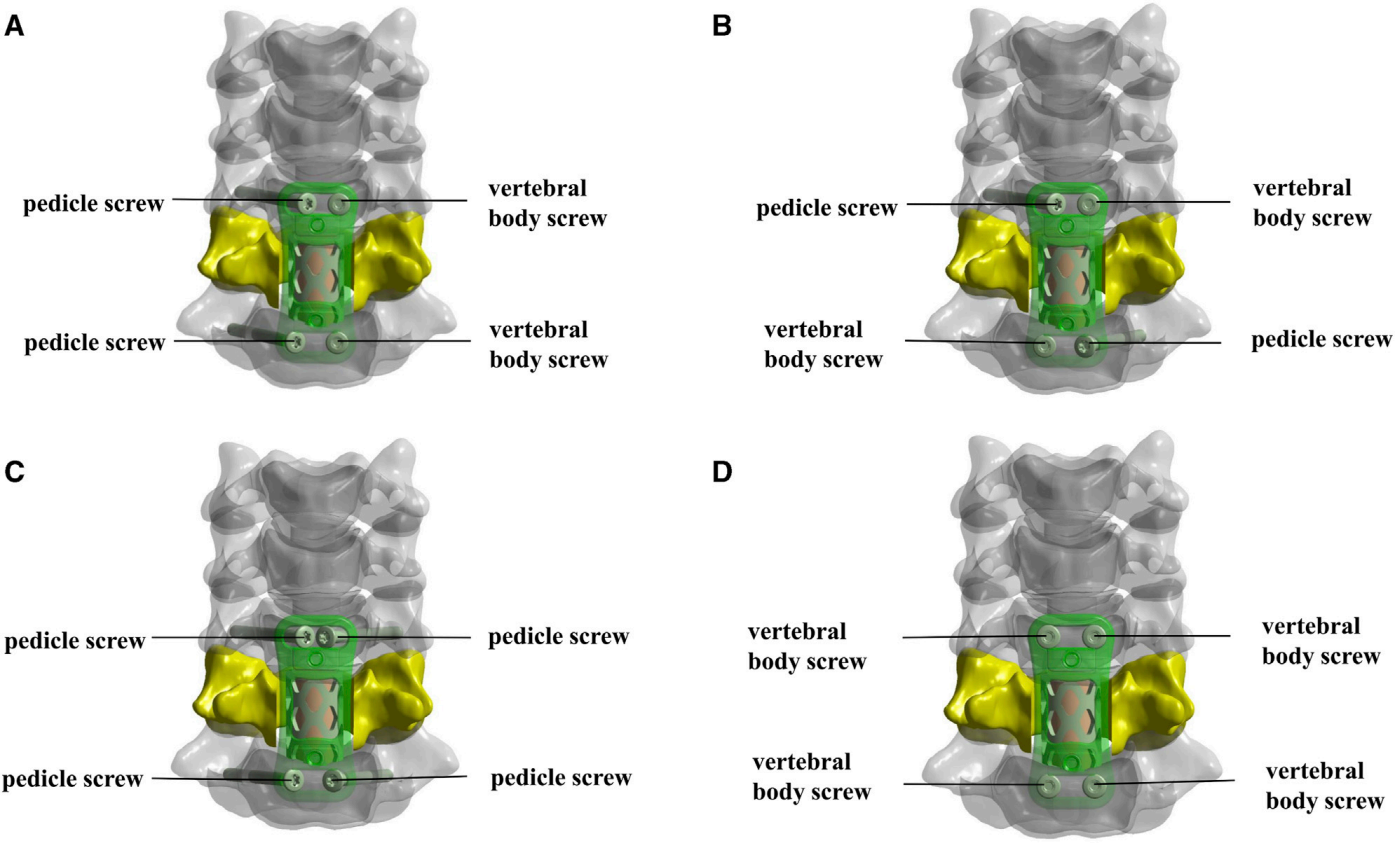
The models of four types of screw instrumentation. **(A,B)** Unilateral ATPS (UATPS): ipsilateral transpedicular screw-contralateral vertebral body screw (ITPS-CVBS, **(A)** and cross transpedicular screw-vertebral body screw (CTPS-VBS, **(B)**; **(C)** bilateral ATPS (BATPS); **(D)** vertebral body screw (VBS).

3D physical modeling was performed for a novel ATPS-plate system of the cervical spine (Patent No: ZL 2018 2 0814089.9) (Figure 4) and for a titanium mesh cage (TMC) by using Solidworks 2015 (Dassault Systemes, France). C6 corpectomy and C5/6 and C6/7 discectomy were simulated, and a TMC packed with cancellous bone was implanted into the decompression groove. The components of the screw-plate system were then assembled. For contact setup: the intervertebral disc, nucleus pulposus, and endplate were bound to each other; the screws and vertebrae contacted inseparably, the screws and plate and the plate and vertebrae contacted in a face-to-face manner. There was separable rough contact between the TMC and endplate, which did not allow for sliding. The finite element model of four types of internal fixation in ACCF following reconstruction for single-level corpectomy and decompression was simulated (Figure 5): UATPS, including ipsilateral transpedicular screw-contralateral vertebral body screw (ITPS-CVBS) and cross transpedicular screw-vertebral body screw (CTPS-VBS); BATPS; and VBS.

The 4-type finite element model was imported into Ansys Workbench 18.0 (ANSYS, United States), and working conditions were established and calculated with reference to the original model of the full set. Subsequently, the ROM, the maximal von Mises stress on TMC, the stress distribution and maximal von Mises stress on screws, and the maximum sliding displacement of screws were analyzed for the four models.

## 3 Results

### 3.1 Validation of C3–C7 vertebral model

**FIGURE 6:**
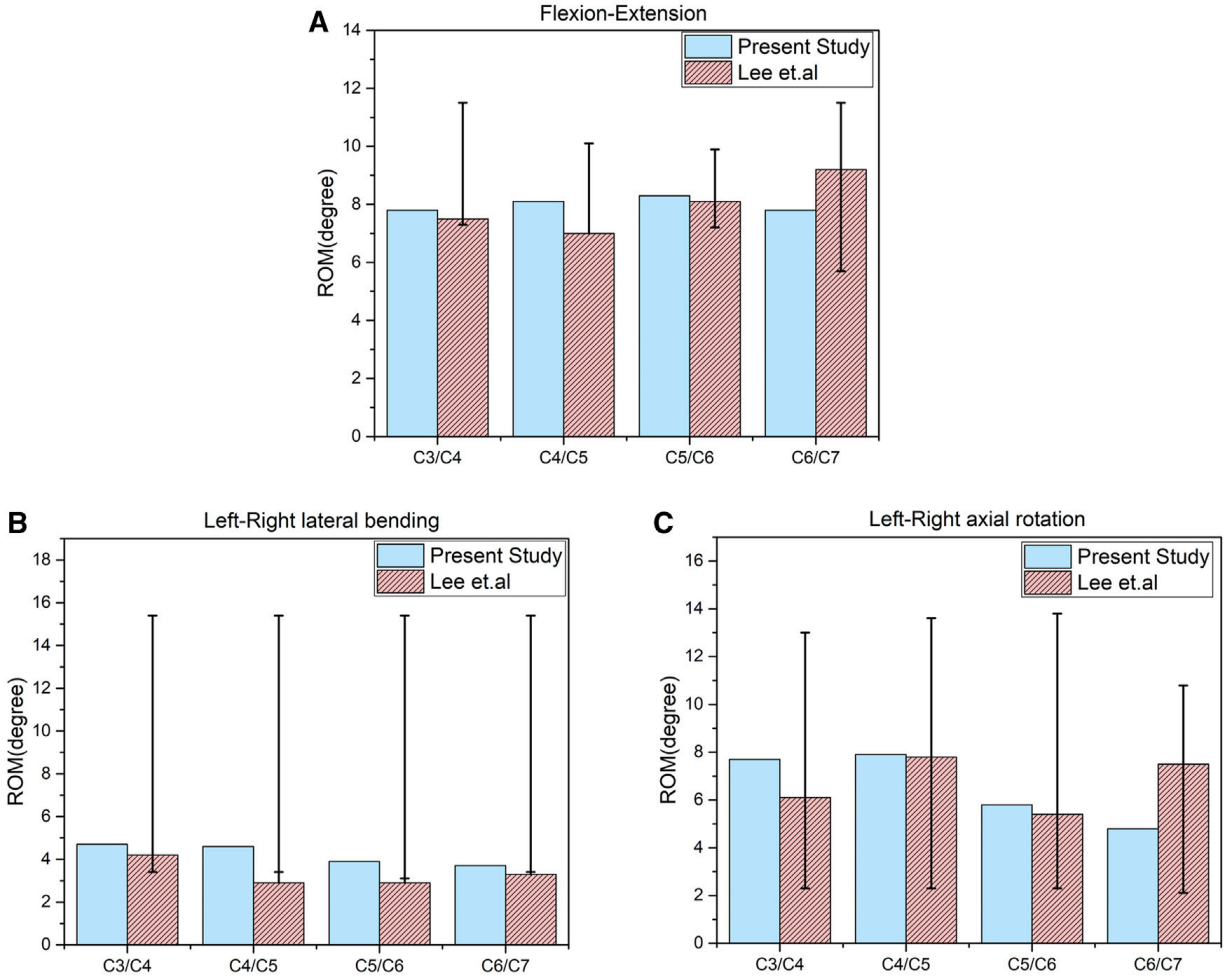
Comparison of the range of motion (ROM) of the original finite element models of C3–C7 with the previous biomechanical study. **(A)** ROM in flexion-extension. **(B)** ROM in lateral bending. **(C)** ROM in axial rotation.

Under pure moments and motion loads, the predicted results of ROM were compared with the results of the validated model to assess the validity of the new model. The results were congruent with the literature (Lee et al., 2011), thus, the model was validated (Figure 6).

### 3.2 Range of motion (ROM)

**FIGURE 7:**
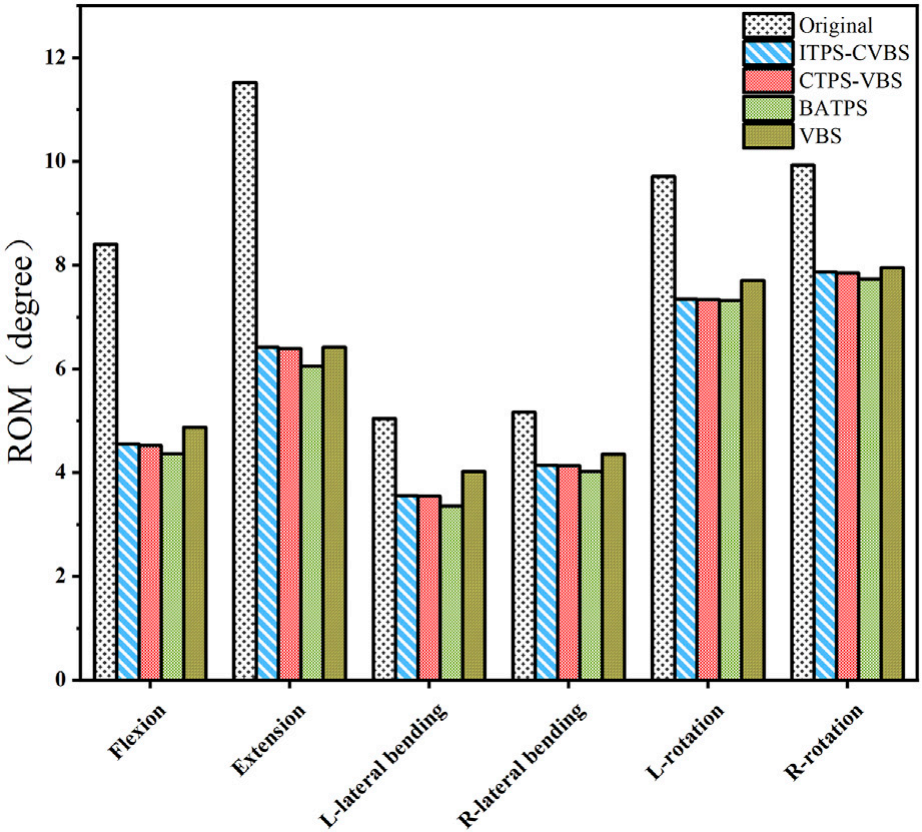
ROM of the different models of fixation.

Compared with the original model, all the internal fixation models exhibited reduced ROM in the six orientations, namely, reduction of ROM by 42.1%–48.1% in flexion, by 44.3%–47.5% in extension, by 20.2%–33.5% in left lateral bending, by 15.7%–22.1% in right lateral bending, by 20.7%–24.6% in left rotation, and by 20.0%–22.2% in right rotation. The ROM differed insignificantly between the four models and was in the ascending order: BATPS<CTPS-VBS<ITPS-CVBS<VBS (Figure 7).

### 3.3 Maximal von Mises stress on TMC

**FIGURE 8:**
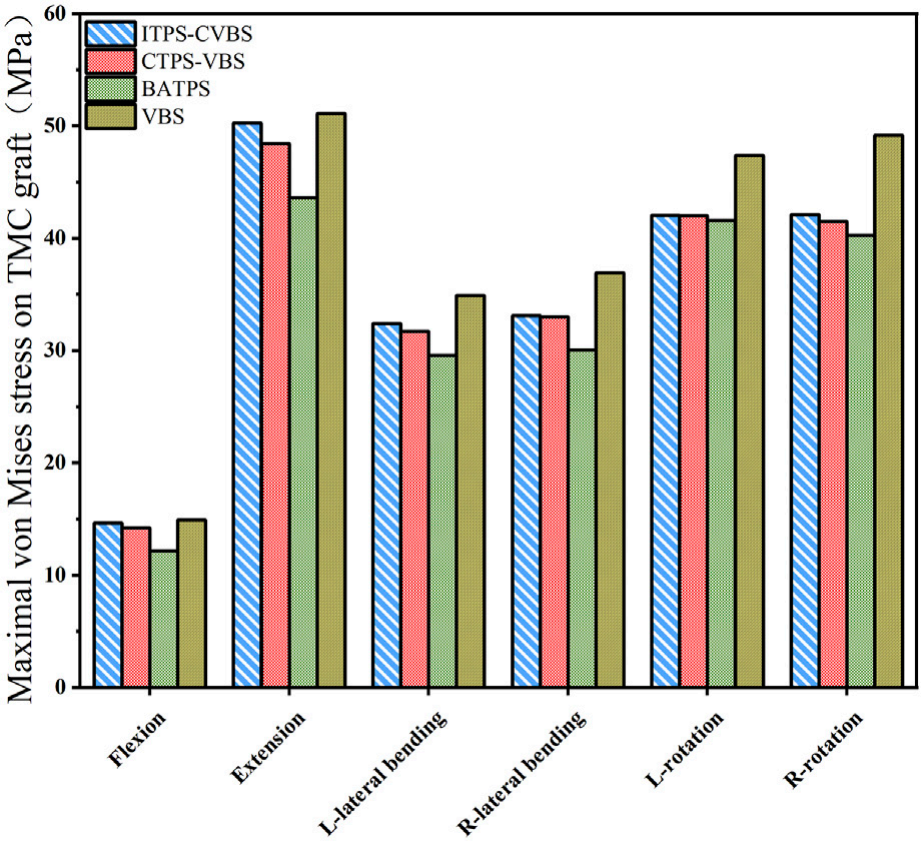
Maximal von Mises stress on TMC graft in the different groups.

Under the loading conditions in flexion, extension, lateral bending, and lateral axial rotation, the maximal von Mises stress on TMC was lowest in the BATPS group (12.18, 43.58, 29.58, 30.05, 51.59, and 40.28 MPa, respectively) and highest in the VBS group (14.92, 51.10, 24.93, 36.92, 47.36, and 49.17 MPa, respectively), while that in the UATPS group was between the BATPS group and the VBS group (CTPS-VBS: 14.20, 48.41, 31.72, 33.00, 41.99, and 41.48 MPa, respectively; ITPS-CVBS: 14.64, 50.26, 32.41, 33.11, 42.03, and 42.09 MPa, respectively) (Figure 8). The stress on BATPS in flexion, extension, and lateral bending was significantly lower than that on UATPS, but the differences were insignificant in rotation. However, regardless of bilateral or unilateral ATPS, the stress on TMC was lower compared with that on VBS.

### 3.4 Maximal von Mises stress and stress cloud map of screws

**FIGURE 9:**
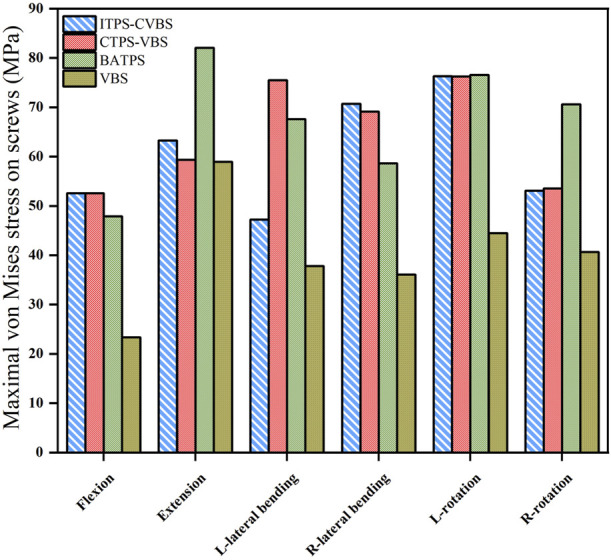
Maximal von Mises stress on screws between different groups.

**FIGURE 10:**
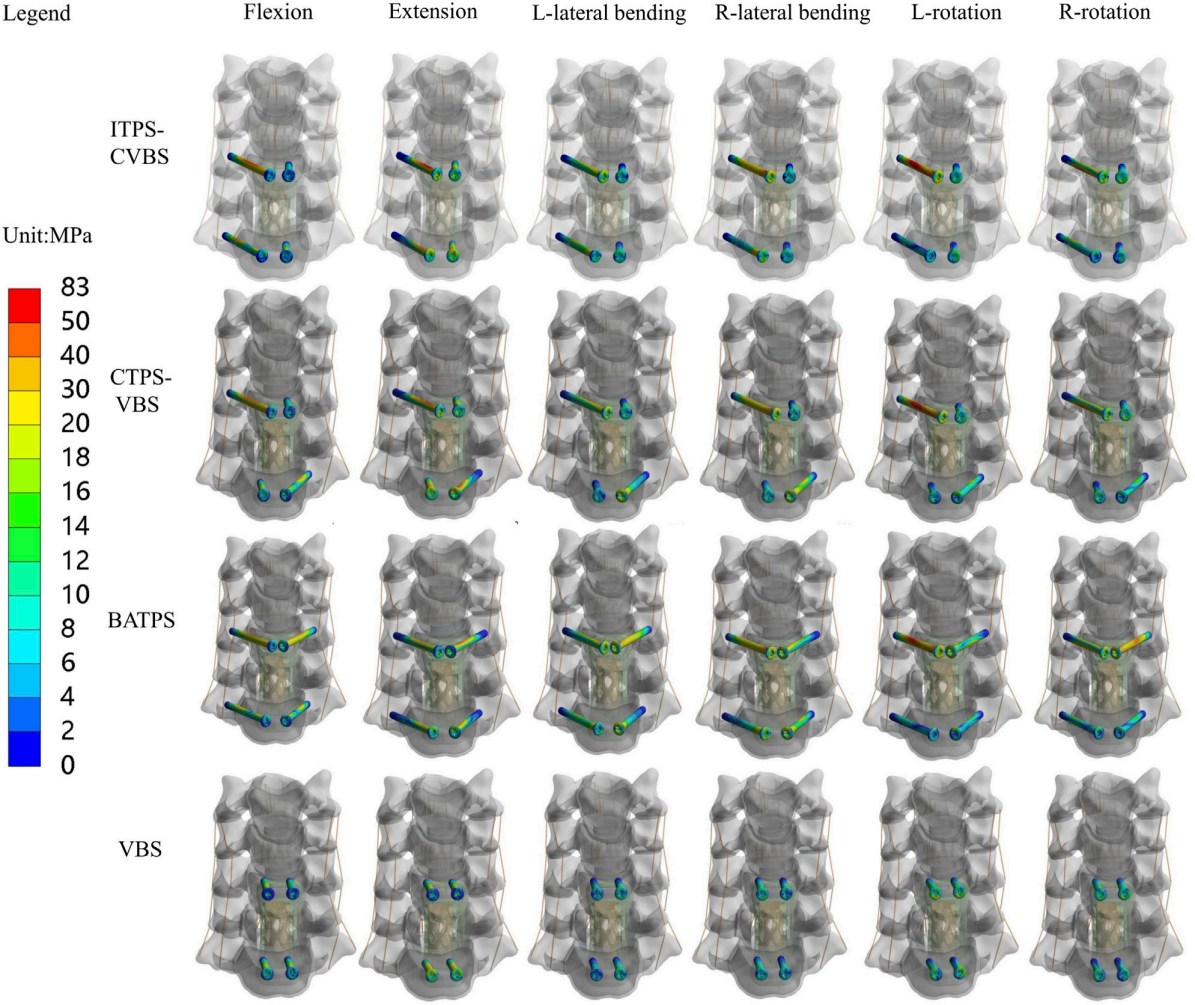
Distribution of the von Mises stress on screws between different groups.

Comparison of UATPS (ITPS-CVBS vs. CTPS-VBS) showed that the stress reduced by 37.4% on ITPS-CVBS when compared to CTPS-VBS in left bending, but the group differences were insignificant in flexion, extension, right lateral bending, and lateral rotation (Figure 9). BATPS endured the maximum stress of 82.07 MPa in extension. VBS endured stress of 23.38, 58.92, 78.80, 36.09, 44.56, and 40.66 MPa, respectively, under the six loads, which were significantly lower than those on transpedicular screws (ITPS-CVBS, CTPS-VBS, and BATPS) (Figure 9). Figure 10 shows the stress distribution on the screws under the six loads in each model group.

### 3.5 Maximum displacement of screws

**FIGURE 11:**
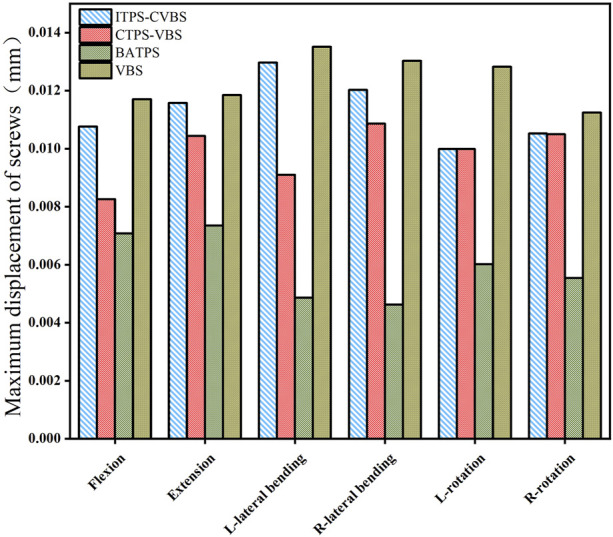
Maximum displacement of screws between different groups.

The maximum displacement of screws was largest in the VBS group under loads in flexion, extension, left and right lateral bending, and left and right lateral axial rotation (Figure 10). When compared with VBS, the maximum displacement of screws reduced by 8.1%, 2.3%, 4.1%, 7.7%, 22.1%, and 6.4%, respectively, with ITPS-CVBS; by 29.4%, 11.9%, 32.7%, 16.6%, 22.1%, and 6.6%, respectively, with CTPS-VBS; and by 39.6%, 37.9%, 64.1%, 64.6%, 53.1%, and 50.7%, respectively, with BATPS (Figure 11).

## 4 Discussion

Following anterior cervical corpectomy, VBS or ATPS can be used for internal fixation. ATPS can anchor the three columns of the vertebra, leading to a higher stability compared with VBS. ATPS technique is insertion pedicle screw from the anterior cervical vertebra, cross-sectional insertion of ATPS into the cervical spine is key to the technique. Koller et al. (2008b) proposed that the ideal cross-sectional entry point for screws is contralateral to the pedicles for C3–C5, but is ipsilateral to the pedicles for C6–T1. Zhao et al. (2018) drew similar conclusions as Koller et al. However, in modeling, we found that the screw into the C5 vertebral body can rotate around the center of the pedicle; thus, the entry point of ATPS was ipsilateral to the pedicles, and the cortical bone of the pedicle was not penetrated (Figure 5C). Therefore, we choose the anterior pedicle screw fixation method should based on the upper and lower cervical corpectomy segment. If the upper vertebral body of the corpectomy segment is C3 or C4, we can only choose one vertebral body screw and one pedicle screw (Figures 5A, B). For C5, one vertebral body screw and one pedicle screw or two pedicle screws can be used. The lower vertebral body of the corpectomy segment is C6 or C7, and two pedicle screws can be inserted (Figure 5C). Hence, we performed C6 corpectomy with screw fixation of C5 and C7, which can meet the requirement of inserting unilateral or bilateral ATPS and VBS and allows for the following biomechanical comparisons.

### 4.1 ROM

Following cervical spinal fusion, a smaller ROM is associated with higher stability and reduced likelihood of loosening of internal fixation devices. Wu et al. (2018) simulated ATPS and VBS internal fixation with six cervical spine specimens and found that ATPS had a smaller ROM than VBS, and that ATPS internal fixation can offer adequate stability for three-column injury to the lower cervical spine. Our results demonstrated that compared with the original model, the four screw insertion methods, namely, BATPS, ITPS-CVBS, CTPS-VBS, and VBS, exhibited significantly reduced overall ROM of the cervical spine under all six loads, with the ROM in the ascending order of ATPS<ITPS-CVBS<CTPS-VBS<VBS (Figure 7). When compared with VBS, ATPS can reduce the ROM of the cervical spine, which is consistent with the findings of Wu et al. (2018). It can be inferred that the use of ATPS can reduce the ROM of the cervical spine and thus increase the stability of the cervical spine, and that such advantages may be more pronounced in the internal fixation for multilevel cervical corpectomy and spinal fusion. Notably, the number of screws used and the method of screw insertion influence the ROM of the cervical spine differently. An increased number of ATPS used is associated with a decreased ROM; given a same number of ATPS or VBS used, cross insertion of ATPS resulted in a smaller ROM and a better overall stability compared with ipsilateral insertion.

### 4.2 Maximal von Mises stress on TMC

Implant displacement and settlement is associated with the stress on the implant-endplate interface. An excess load of the endplate may lead to implant displacement and endplate damage, ultimately resulting in failure of the internal fixation. In this study, the maximal von Mises stress on TMC was lowest in flexion and highest in extension in each group, possibly because the screws and plate in front of the cervical spine can offset some stress in flexion. However, the magnitude of the stress varied among the different methods of fixation. Under various working conditions in motion, the maximal von Mises stress on TMC was lowest with BATPS, was lower with CTPS-VBS than with ITPS-CVBS, and was highest with VBS. Hence, internal fixation with VBS yields greater stress on TMC and is likely to cause damage to the endplate bone.

### 4.3 Maximal von Mises stress and stress cloud map of screws

Owing to bending, deformation, and loosening, the anchoring components and rods between the screw and bone are liable to displacement (Oda et al., 2022). Fogel et al. (2003) also reported that fracture is likely to occur in the presence of failure between the screwhead and screw. In the clinical setting, the screw is usually fractured at the junction of the nut and the plate, and the stress on this junction is key to facture of the screw. In this study, the stress cloud map showed that the stress was predominantly concentrated at a point two-thirds of the length from the tail of the screw after ACCF, the stress of the ATPS from the cranial part was higher than that of the caudal part. The maximal von Mises stress was greater on unilateral or bilateral ATPS compared with that on VBS. VBS inserted into the anterior and middle columns of the vertebral body and into the cancellous bone of the vertebral body can bear a small stress during movements of the cervical spine. In contrast, ATPS penetrates the anterior, middle, and posterior columns of the vertebral body; hence, it bears a great stress during movements of the cervical spine, thereby avoiding loosening. The screw stress cloud maps did not show any red areas reflective of a concentration of the stress on VBS in the six orientations of movement, possibly because of transfer of the stress onto the TMC. Hence, the likelihood of TMC displacement is high with VBS. With respect to UATPS fixation, the stress was reduced by 37.4% with ITPS-CVBS when compared with CTPS-VBS in left bending, possibly because no ATPS shared the stress in ITPS-CVBS at the left side of the cervical spine. Hence, in light of the maximal von Mises stress on screws and the stress cloud map, ATPS can tolerate greater stress than VBS during cervical spine movements. BATPS can balance the stress during cervical spine movements better than UATPS. In terms of UATPS, CTPS-VBS can tolerate lateral bending better than ITPS-CVBS.

### 4.4 Maximum displacement of screws

The maximum displacement of the screw is proposed as a stability parameter (Li et al., 2013), which can reflect the overall stability of the screw-plate system better than the ROM of the cervical spine and the stress on internal fixation devices. Screw loosening results from insufficient stress on the screw-bone interface, and the bone density of the vertebral body, the length of screw, the thread type, the screw diameter, and single or double cortical fixation all influence screw stability (Zhang et al., 2006; Matsukawa et al., 2016). Pedicle is the most abundant area of cortical bone in the cervical spine. Koller et al. (2008a) demonstrated that the fixation strength of ATPS was 2.5 times that of conventional anterior VBS. In this study, under loads in flexion, extension, lateral bending, and lateral axial rotation, the maximum displacement was largest with VBS, smallest with BATPS, and was moderate with ITPS-CVBS and CTPS-VBS. UATPS showed even smaller displacement than VBS under the six loads, suggesting a good stability, which is consistent with the literature (Koller et al., 2010; Zhao et al., 2018). With respect to UATPS, CTPS-VBS exhibited smaller screw displacement and better stability compared with ITPS-CVBS in flexion, extension, and lateral bending, but not in rotation. Hence, in terms of the ATPS technique, stability is highest with BATPS, followed by CTPS-VBS, and is lowest with ITPS-CVBS.

Taken together, based on the ROM, the maximal von Mises stress on TMC, the stress distribution and maximal von Mises stress on screws, and the maximum sliding displacement of screws, we compared different methods of anterior screw insertion into the cervical spine in ACCF and demonstrated that the stability was highest with BATPS, followed by CTPS-VBS, ITPS-CVBS, and then VBS. Hence, the ATPS technique can reduce the incidence of screw loosening and TMC displacement.

### 4.5 Limitations of the study

This study does have some limitations. The study investigated the initial stability of different screw fixations following single-level ACCF, and further studies need to be conducted on the ultimate mechanical properties and fatigue resistance of these screw fixations. In addition, no finite element model involves muscles, and the data may change with the addition of muscles. Furthermore, the model in this study did not simulate multilevel ACCF or osteoporosis-related working conditions. Our data are expected to change in multilevel spinal fusions and osteoporosis, but the overall trend of stability may not change. In addition, *in vitro* biomechanical testing and clinical studies need to be performed to appraise the results of this study.

### 4.6 Conclusion

The stability of various methods of anterior screw insertion in the cervical spine for ACCF differs. The findings of this study hold the potential to aid in the development of an optimal fixation method for lower cervical spinal fusions, with the goal of reducing internal fixation failures following anterior cervical spinal fusions. In the context of finite element analysis (FEA), BATPS is recommended whenever feasible, and in cases where only UATPS insertion is possible, CTPS-VBS is recommended. Prior to conducting clinical trials, it remains essential to perform *in vitro* experiments to further analyze and validate the results.

## Data Availability

The original contributions presented in the study are included in the article/Supplementary Material, further inquiries can be directed to the corresponding authors.
